# Comparison of apixaban and rivaroxaban for anticoagulant effect after lumbar spine surgery: a single-center report

**DOI:** 10.4155/fsoa-2017-0123

**Published:** 2018-03-14

**Authors:** Kun Zhang, Shenghao Zhao, Wusheng Kan, Jun Xiao, Feifei Pu, Kun Li

**Affiliations:** 1 Department of Orthopaedics, Wuhan Puai Hospital, Affiliated to Huazhong University of Science & Technology, Wuhan 430033, PR China; 2 Department of Orthopedics, Wuhan No.1 Hospital, Wuhan Integrated TCM & Western Medicine Hospital, Wuhan 430022, PR China

**Keywords:** apixaban, lumbar spine surgery, rivaroxaban, venous thromboembolism

## Abstract

**Aim:**

To compare efficacy and safety of anticoagulants apixaban and rivaroxaban after lumbar spine surgery retrospectively.

**Methods:**

In this study comprising 480 patients, lower-limb swelling, deep venous thrombosis, pulmonary embolism, blood loss, ecchymosis and D-dimer changes were observed.

**Results:**

The changes in perimeter of the legs were tested, and significant differences were noted 10 cm above the patella, but no significant differences 10 cm below the patella. Twelve thrombotic events occurred in the apixaban group and nine in rivaroxaban group. No significant difference in the incidences of thromboembolic events (p = 1.00). Compared with rivaroxaban, there was significantly less bleeding in the apixaban group (p = 0.03).

**Conclusion:**

Apixaban and rivaroxaban were equally effective anticoagulation therapies that exhibited similar preventive effects against postoperative venous thromboembolism after lumbar spine surgery.

## Lay abstract

Venous thromboembolism (VTE) can lead to severe morbidity with poor quality of life and even sudden death related to pulmonary embolism. VTE risk factors are common in patients with degenerative spine, and without prophylaxis, approximately 15% of patients undergoing posterior spinal surgery develop deep vein thrombosis. In this study, efficacy and safety of anticoagulants apixaban and rivaroxaban after lumbar spine surgery were compared retrospectively. This study confirmed that apixaban and rivaroxaban were equally effective anticoagulation therapies that exhibited similar preventive effects against postoperative VTE after lumbar spine surgery.

Venous thromboembolism (VTE) is a common and potentially lethal disease that comprises deep vein thrombosis (DVT) and pulmonary embolism (PE). VTE can lead to serious illness, low quality of life and even sudden death from sports related to PE. About half of all untreated DVT cases are caused by PE; In contrast, 50–80% of untreated PE cases are associated with DVT [1]. VTE is known about with advanced age, smoking, obesity, major surgery, hospitalization, immobilization, neurological deficit, blood transfusion, malignancy, trauma, an inherited hypercoagulable state and oral contraceptive use [2].

In spinal surgery, the factors of venous stasis are long-time horizontal ventral decubitus, lack of muscle tone, venous compression by retractors and postoperative bed rest. Venous intimal injury may occur in surgical handling [3]. VTE risk factors are common in patients with degenerative spine, and without prophylaxis, approximately 15% of patients undergoing posterior spinal surgery develop DVT [4]. The risk can be lowered with mechanical devices that provide intermittent pneumatic compression, but dual mechanical/pharmacologic prophylaxis is more effective [5]. Despite this risk and the superiority of dual prophylaxis, pharmacologic prophylaxis was routinely used by only 31% of recently surveyed orthopedic spine surgeons [6].

Apixaban and rivaroxaban, both novel oral anticoagulants, have been clinically used following orthopedic surgery, particularly for preventing VTE after hip and knee replacement surgery [7,8]. Although these two novel oral anticoagulants have been proven safe and effective, no comparative report has described the efficacy and safety of its anticoagulation ability after spinal surgery. Thus, the objective of this study is to compare the effectiveness and safety of apixaban and rivaroxaban for preventing VTE after lumbar spine surgery.

## Methods

### Ethics statement

This study was approved by the ethics committee of Puai Hospital, Tongji Medical College, Huazhong University of Science and Technology. Informed consent was obtained from each patient.

### Patients

This retrospective study included patients who underwent spinal operations at the Department of Spinal Surgery, Puai Hospital, Tongji Medical College, Huazhong University of Science and Technology in China between May 2015 and 2017. These patients were divided into apixaban and rivaroxaban groups randomly. The inclusion criteria were complete medical records including patient number, sex, age, bodyweight, body height, regional distribution, admission date, length of hospital stay, occupation, lower-extremity ultrasonography findings, DVT, spinal epidural hematoma, hypertension, diabetes, heart disease, surgical method, level and number of vertebrae fused, operative duration, blood loss and blood transfusion (Table 1). The exclusion criteria were conservative treatment and percutaneous vertebroplasty, since the patients were discharged soon from hospital after percutaneous vertebroplasty. In addition, patients were excluded if they suffered from preoperative DVT or ever took an anticoagulant such as warfarin, aspirin or clopidogrel during the week prior to the hospital admission.

**Table 1.** **Patient characteristics.**

**Items**	**Apixaban group**	**Rivaroxaban group**	**p-value**
Patient number (n)	240	240	1

Male/female (n)	157/83	135/105	0.015

Age (year)	69 ± 12	64 ± 8	0.391

Bodyweight (kg)	61 ± 16	68 ± 17	0.721

Body height (cm)	168 ± 11	172 ± 9	0.093

Hospital stay (day)	7.9 ± 0.8	8.4 ± 0.6	0.029

### Study protocol

The surgical approaches included thoracolumbar discectomy, laminectomy and fixation. The decision to use apixaban or rivaroxaban was not at the surgeons’ discretion or a policy relating to differing use based on surgery type. Patients in the apixaban group were prescribed 2.5 mg orally twice daily for 14 days commencing at 8 am the morning following surgery. Patients in the rivaroxaban group began daily oral treatment with 10 mg 6–8 h after surgery and the treatment continued until the 14th day, when the patients could fully ambulate. Physical and pharmacological methods of antithrombotic prophylaxis other than dalteparin and concomitant treatment with aspirin, other antiplatelet agents or nonsteroidal anti-inflammatory drugs were not permitted during the trial.

All patients were prescribed graduated compression stockings for 6 weeks, and calf-length intermittent pneumatic compression devices while in the hospital, and encouraged to mobilize on the first day after lumbar spine surgery. Bilateral lower-limb Doppler ultrasonography screening for VTE was performed between postoperative day 3 and 7. Diagnostic tests for PE were only performed if clinically indicated. Routine blood tests were performed preoperatively, day 1 postoperatively and before discharge.

### Outcome assessment

All of the patients were assessed daily during the hospital course to review their clinical status, including symptoms and signs of VTE, bleeding side effects and other adverse events including swelling, pain, rigidity, differences in calf diameter, Homan's sign, warmth, tenderness and erythema. Patients with clinically suspected DVT underwent color Doppler sonography and those with clinical features suggestive of PE underwent a chest perfusion scan. The participants were followed up by hospital visits, telephone calls or office visits until 90 days after surgery to document the occurrence of clinically overt VTE, bleeding, other side effects or death. Patients with positive sonographic and perfusion scan results were treated with unfractionated heparin and warfarin. Acute DVT diagnosed in this study was classified by anatomic location.

### Statistical analysis

SPSS 20.0. software was used for the statistical analysis. Student's *t*-test was used to compare perimeter changes of the legs, bleeding and D-dimer level changes. The χ^2^ test was used to compare the incidences of thromboembolic events between the two groups. If the number of cases was ≥5, then the χ^2^ test was used. If the number of cases was <5, then the corrected χ^2^ test was used. If the number of cases was ≤1, then Fisher's exact test was used. p < 0.05 was considered statistically significant.

## Results

In the apixaban group and rivaroxaban group, the perimeter changes of legs were tested; there were significant differences in both left and right legs 10 cm above the patella (p < 0.05) (Table 2). But, the perimeter changes exhibited no significant differences of both left and right legs  10 cm down the patella (p > 0.05) (Table 2).

**Table 2.** **The perimeter changes of legs (patellar 10 cm).**

**Items**	**Up patellar (left)**	**Up patellar (right)**	**Down patellar (left)**	**Down patellar (right)**
Apixaban	2.33 ± 0.09	2.3 ± 0.09	1.43 ± 0.1	1.4 ± 0.06

Rivaroxaban	2.17 ± 0.06	2.14 ± 0.05	1.34 ± 0.11	1.36 ± 0.08

p-value	0.02	0.009	0.14	0.1

In the apixaban group, 12 thrombotic events occurred (5% of cases). Two patients died of PE, and two patients exhibited nonfatal PE. One patient exhibited femoral vein thrombosis, floating blood clots and a swollen leg. The other patient demonstrated external iliac vein thrombosis, a swollen leg, difficulty breathing and a progressive decline in arterial oxygen pressure. Totally, six patients exhibited DVT by color Doppler sonography. In the rivaroxaban group, nine thrombotic events occurred (3.75% of cases). One patient died of PE and two exhibited nonfatal PE. One symptomatic VTE patients exhibited femoral vein thrombosis, inferior vena caval filter insertion was taken. Totally, five patients exhibited DVT by color Doppler sonography. No significant intergroup difference was found in the incidences of thromboembolic events between apixaban and rivaroxaban (p > 0.05) (Table 3).

**Table 3.** **Occurrence rate of thrombus.**

**Items**	**Apixaban group (n/N)**	**Rivaroxaban group (n/N)**	**p-value**
Death	2/240 (0.83%)	1/240 (0.42%)	1

Nonfatal PE	2/240 (0.83%)	2/240 (0.83%)	1

DVT	6/240 (2.5%)	5/240 (2.08%)	1

Symptomatic VTE	2/240 (0.83%)	1/240 (0.42%)	1

DVT: Deep vein thrombosis; PE: Pulmonary embolism; VTE: Venous thromboembolism.

In the apixaban group, mean total bleeding was 1397 ± 99 ml and mean invisible bleeding was 842 ± 17 ml; in the rivaroxaban group, mean total bleeding was 1535 ± 77 ml and mean invisible bleeding was 855 ± 22 ml. Compared with rivaroxaban, total bleeding and invisible bleeding were significantly lower in the apixaban group (p < 0.05) (Table 4).

**Table 4.** **Occurrence of bleeding (ml).**

**Items**	**Total bleeding**	**Invisible bleeding**
Apixaban	1397 ± 9	9842 ± 17

Rivaroxaban	1535 ± 77	855 ± 22

p-value	0.03	0.02

Postoperative D-dimer level changes (postoperative 1, 3, 7 and 14 days) were lower in the apixaban group than in the rivaroxaban group, and there were significant intergroup differences in bleeding (p < 0.05) (Table 5).

**Table 5.** **The changes of D-dimer (mg/l).**

**Items**	**Postoperation 1d**	**Postoperation 3d**	**Postoperation 7d**	**Postoperation 14d**
Apixaban	2.14 ± 0.01	3.28 ± 0.14	4.24 ± 0.1	5.32 ± 0.13

Rivaroxaban	2.16 ± 0.02	3.46 ± 0.18	4.51 ± 0.13	5.6 ± 0.11

p-value	0.046	0.014	0.009	0.009

## Discussion

The objective of the North American Spine Society Evidence-Based Clinical Guideline on antithrombotic therapies in spinal surgery was to provide evidence-based recommendations to address key clinical questions surrounding the use of therapies in spinal surgery [9]. The goal of the guideline's recommendations was to assist with delivering optimum efficacious treatment and prevent thromboembolic events.

Major elective spine surgery is a recognized risk factor for VTE, and PE is a significant cause of morbidity and mortality in this patient population despite the use of traditional prophylactic methods. The risk of VTE can be lowered with the use of mechanical and pharmacologic prophylaxis. Mechanical interventions include elastic compression stockings and intermittent pneumatic compression devices [1,2]. Practical limitations of the current prophylactic techniques have stimulated a search for simpler methods. Low-molecular-weight heparins and fondaparinux require subcutaneous injections.

The development of new oral anticoagulant agents has raised hopes that they will combine greater convenience with efficacy and safety profiles that are similar to or better than those of other methods. The pharmacological mechanism of rivaroxaban involves directly antagonizing blood coagulation factor X to leverage its anticoagulation properties [7–9]. Rivaroxaban exhibits high oral bioavailability. However, reports describing its efficacy and postoperative safety assessment after spinal surgery are rare. The use of rivaroxaban, a factor Xa inhibitor, a direct thrombin inhibitor, to prevent VTE after joint replacement surgery has been evaluated in several Phase III clinical trials [10]. Apixaban, a highly specific factor Xa inhibitor, is administered as a twice daily fixed dose and does not require routine laboratory monitoring. Clinical trials of apixaban involving patients who underwent elective knee replacement surgery showed that, compared with enoxaparin, apixaban had better efficacy with a similar or lower risk of bleeding [11].

Our study shows efficacy and safety of anticoagulants, apixaban and rivaroxaban after lumbar spine surgery were compared retrospectively. This study confirmed that apixaban and rivaroxaban were equally effective anticoagulation therapies that exhibited similar preventive effects against postoperative VTE after lumbar spine surgery. In a meta-analysis of six randomized clinical trials, they compared the efficacy and safety of direct factor Xa inhibitors (rivaroxaban and apixaban) with enoxaparin for the prevention of VTE after total knee replacement. Their results confirmed that direct Xa inhibitors (rivaroxaban and apixaban) were more effective for prevention of VTE after total knee replacement as compared with enoxaparin, without increasing major bleeding risk [12]. This conclusion is consistent with our findings; our study suggests that the new oral anticoagulant (rivaroxaban and apixaban) may have good safety and efficacy in preventing DVT after spine surgery, which will provide more benefit for patients after lumbar spine surgery.

Our study has limitations. First, the conclusions are based on an analysis of retrospective data. Second, the data do not allow assessment of clinician-specific decision making. Also, the sample size was small; it was conducted at a single spine center potentially introducing selection bias.

## Conclusion & future perspective

Combined mechanical/pharmacologic prophylaxis should be considered in spinal surgery patients. Apixaban and rivaroxaban are new oral anticoagulant agents with documented efficacy and postoperative safety after spinal surgery. Apixaban has the advantage of reduced bleeding compared with rivaroxaban.

Larger prospective studies are required to more definitively assess the efficacy and safety of apixaban and rivaroxaban. This requires the verification of bleeding risk and other adverse effects in further animal experiments and clinical trials. In addition, the specific antagonist of the new oral anticoagulant drug is also in the clinical trial stage, so it is reasonable to expect that in the future, the further development of new oral anticoagulants will definitely bring more benefits for both doctors and patients.

**Executive summary**

- Compared efficacy and safety of anticoagulants apixaban and rivaroxaban after lumbar spine surgery retrospectively.
- Both of apixaban and rivaroxaban are new oral anticoagulant agents with efficacy and postoperative safety after spinal surgery.
- Apixaban has the advantage of reduced bleeding compared with rivaroxaban, it was recommended as the first choice anticoagulant after lumbar spine surgery.
